# Cross‐stress tolerance: Mild nitrogen (N) deficiency effects on drought stress response of tomato (*Solanum lycopersicum* L.)

**DOI:** 10.1002/pei3.10060

**Published:** 2021-10-07

**Authors:** Vajiheh Safavi‐Rizi, Kora Uellendahl, Britta Öhrlein, Hamid Safavi‐Rizi, Christine Stöhr

**Affiliations:** ^1^ Department of Plant physiology Institute of Botany and Landscape Ecology University of Greifswald Greifswald Germany; ^2^ Department of Information Technology Engineering Institute of Information Technology and Computer Engineering University of Payame noor Isfahan Iran

**Keywords:** climate change, cross‐stress tolerance, drought, nitrogen deficiency, tomato

## Abstract

Climate change will lead to more frequent and severe drought periods which massively reduce crop production worldwide. Besides drought, nitrogen (N)‐deficiency is another critical threat to crop yield production. Drought and N‐deficiency both decrease photosynthesis and induce similar adaptive strategies such as longer roots, reduction of biomass, induction of reactive oxygen species (ROS), and antioxidative enzymes. Due to the overlapping response to N‐deficiency and drought, understanding the physiological and molecular mechanisms involved in cross‐stresses tolerance is crucial for breeding strategies and achieving multiple stress resistance and eventually more sustainable agriculture. The objective of this study was to investigate the effect of a mild N‐deficiency on drought stress tolerance of tomato plants (*Solanum lycopersicum* L., cv. Moneymaker). Various morphological and physiological parameters such as dry biomass, root length, water potential, SPAD values, stomatal conductance, and compatible solutes accumulation (proline and sugar) were analyzed. Moreover, the expression of ROS scavenging marker genes, cytosolic *ASCORBATE PEROXIDASES* (*cAPX1*, *cAPX2*, *and cAPX3*), were investigated. Our results showed that a former mild N‐deficiency (2 mM NO_3_^−^) enhances plant adaptive response to drought stress (4 days) when compared to the plants treated with adequate N (5 mM NO_3_^−^). The improved adaptive response was reflected in higher aboveground biomass, longer root, increased specific leaf weight, enhanced stomatal conductance (without reducing water content), and higher leaf sugar content. Moreover, the *APX1* gene showed a higher expression level compared to control under N‐deficiency and in combination with drought in the leaf, after a one‐week recovery period. Our finding highlights a potentially positive link between a former mild N‐deficiency and subsequent drought stress response in tomato. Combining the morphological and physiological response with underlying gene regulatory networks under consecutive stress, provide a powerful tool for improving multiple stress resistance in tomato which can be further transferred to other economically important crops.

## 1 INTRODUCTION

Drought, due to climate change, and N‐deficiency are arguably two major threats for agriculture resulting in large economic loss due to reduced yield (Lupini et al., 2017; Safavi‐Rizi et al., 2018).

N is one of the nutrients needed in high quantity and is essential for the synthesis of macromolecules such as nucleic acids, proteins as well as secondary metabolites (Qin et al., 2019). N is a limiting factor for plant growth and development (Coruzzi & Bush, 2001). In agriculture, this limitation is overcome by the application of N‐fertilizers causing negative economic and environmental effects such as eutrophication and global warming (Gruber & Galloway, 2008; Qin et al., 2019). Optimizing N uptake and N remobilization in plants is essential for sustainable agriculture and minimizing the negative environmental and economic consequences of excess N‐fertilizer application (Koeslin‐Findeklee et al., 2015; Safavi‐Rizi et al., 2018). It is estimated that a 1% increase in N use efficiency (NUE) can save about $1.1 billion annually in global agriculture (Kant et al., 2011). Therefore, it is crucial to understand the plant response mechanisms to low N condition for improving NUE (Safavi‐Rizi et al., 2018). N‐deficiency results in various physio‐morphological changes in plants such as longer roots, enhanced nutrient remobilization (in particular for N), increased cell wall lignification, higher activity of antioxidative enzymes (e.g., peroxidases) and accumulation of osmolytes and secondary metabolites (Gross et al., 2013; Kusano et al., 2011).

Under N‐deficiency, ROS level has been reported to increase in certain areas of the root indicating its importance for response to N‐deficiency (Nieves‐Cordones et al., 2019; Shin et al., 2005). Using ascorbate as an electron donor, ascorbate peroxidase (APX), which uses hydrogen peroxide (H_2_O_2_) as substrate, catalyzes its reduction to H_2_O in different cell compartments (Sofo et al., 2015). cAPX1 has been shown to play a key role in regulating the ascorbic acid (AsA) metabolism under N‐deficiency in the tea plant (Li et al., 2020).

Drought stress causes growth inhibition, especially the growth of leaves and stems (Zhang et al., 2019). In contrast, root growth is promoted when water availability is restricted to facilitate water uptake from the soil (Wang et al., 2017). Another adaptation strategy to drought is the regulation of the osmotic potential by accumulating compatible solutes, which are osmotically active but not interfering with metabolic processes (Morgan, 1984; Wani et al., 2013). A variety of compounds for osmoregulation has been found to be accumulated under drought, such as sugars, sugar alcohols and amino acids such as proline, but also hydrophilic proteins (Hartmann & Trumbore, 2016).

Furthermore, ROS elevation due to the reduced photosynthesis is a consequence of drought stress (Hasanuzzaman et al., 2020). The balance between ROS generation and scavenging is therefore essential for drought stress tolerance (Cruz de Carvalho, 2005). A positive correlation between the expression and activity of APX under drought‐induced oxidative stress has been reported in finger millet (Bartwal & Arora, 2016). Moreover, in the former study, a higher APX expression level was observed in the drought‐tolerant variety (PR202) compared to the drought‐sensitive one (PES400) (Bartwal & Arora, 2016).

Similar physiological responses are linked to both N‐deficiency and drought stress response. Drought and N‐deficiency both decrease photosynthesis and induce similar adaptative strategies such as longer root, accumulation of osmolytes, reduction of stomatal conductance and photosynthesis, induction of cell‐damaging ROS species and induction of antioxidative enzymes such as peroxidases. Therefore, a former mild N‐deficiency might improve drought stress tolerance. Former studies have shown that there is a positive correlation between plant water status and N uptake (Tilling et al., 2007). Managing both, water availability and N supply is therefore important for sustainable agriculture (Zillmann et al., 2006). Despite the frequent occurrence of both stresses in the field, the effect of N‐deficiency on the tolerance of plants toward subsequent drought stress has not yet been addressed.

Tomato is an important vegetable crop, and its yield and quality are highly affected by various abiotic stresses (Gerszberg & Hnatuszko‐Konka, 2017; Klay et al., 2018; Safavi‐Rizi et al., 2020a, 2020b). Several former studies have investigated the effect of drought on the response of tomato plants (Patane et al., 2016; Tamburino et al., 2017; Zhou et al., 2019). Here, we investigated the effect of N‐deficiency on drought stress tolerance in tomato (cv. Moneymaker). It has been reported that in *Solanum penellii*, the wild relative of cultivated tomato, drought stress affects the expression of several key genes involved in N metabolism such as glutamate dehydrogenase 2 (GDH2) and asparagine synthetase 1 (ASN1) which play a role in the glutamate and asparagine biosynthesis, respectively. Moreover, genes involved in N assimilation, glutamine oxoglutarate aminotransferase/glutamate synthase (GOGAT/GS) cycle, and γ‐aminobutyric acid (GABA)‐shunt as well as jasomonic acid and ethylene biosynthesis and signaling pathways were differentially regulated in wild tomato compared to cultivated tomato. This indicates the importance of N metabolism and N‐related pathways in drought tolerance (Egea et al., 2018).

The objective of this study was to investigate the impacts of a former mild N‐deficiency on subsequent drought stress tolerance in tomato at the vegetative stage. We examined whether a former mild N‐deficiency might be beneficial for subsequent drought stress tolerance through similar morphological, physiological and to some extent ROS scavenging response between both stresses.

## 2 MATERIAL AND METHODS

### 2.1 Plant growth and treatment

All experiments were performed with *Solanum lycopersicum* (cv. Moneymaker). The tomato seeds were provided by the company “Saatzucht Bardowick GmbH.” The plants were grown in the greenhouse of the Institute of Botany and Landscape Ecology of the University of Greifswald with 500 μmol photons/m2/s and 25℃ under a 16/8‐h light/dark regime. Tomato seeds were sown in 40 small plastic pots made of polypropylene filled with quartz sand (Quartzwerke GmbH, Werferlingen). The sand was a mixture with two‐thirds of rough‐grained (0.7–1.2 mm) and one‐third of fine‐grained sand (0.1–0.5 mm). The pots were placed in plastic trays containing modified Hoagland nutrient solution with 5 mM NO_3_^−^ as described previously (Safavi‐Rizi et al., 2020a, 2020b), to keep the sand moist. An equal amount of nutrient solution was given to the trays daily in the morning.

### 2.2 Stress treatment and experimental set‐up

16 days after sowing the seeds, half of the plants from the experiment were treated with N‐deficiency (2 mM NO_3_^−^) for five days. The other half of the plants (control) continued to be treated with 5mM NO_3_^−^ in the nutrient solution. Following this phase, all plants were treated with 5mM NO_3_^−^, allowing the stressed plants to recover for 2 days. After this recovery phase, four plants of each treatment were harvested and analyzed (Figure S1). The remaining 16 plants per treatment were then divided randomly. Eight plants from each treatment were put under drought stress, water withdrawal, for four days. The control plants received nutrient solution. After four days of drought stress, four plants per treatment were harvested and analyzed (Figure S1). The remaining 16 plants were treated with the standard nutrient solution for the following seven days and analyzed in the end (Figure S1).

### 2.3 Trial series of different drought durations

Twenty plants grown in the pots were treated similarly to the plants in the main experiment. At 14 days after sowing (DAS), four pots were transferred to a dry tray and left under drought for seven days. Three days later, four other pots were transferred to a dry tray for four days (Figure S2). Two days after that, another four pots were put under drought for two days. Afterward, all plants plus four control plants, that had not experienced drought, were analyzed (Figure S2). All plants had the same age at the time of the harvest. The whole plants were carefully removed from the sand and the fresh biomass was measured on the micro scales (BP 160 P, Sartorius). The plants were then put in coffee filter bags and dried in a dry cabinet (Series FD Classic Line, Binder) at 70°C for 48 h for dry weight assessments. The water content (%) was calculated based on the difference between fresh and dry weight relative to fresh weight * 100. The same procedure was conducted with the sand.

### 2.4 Measurements of specific leaf weight

A cork borer with a diameter of 0.8 cm was used to punch out two pieces of the oldest leaf above the cotyledons. Those pieces were then weighed together on the micro scales (BP 160 P, Sartorius). All samples were put in coffee filter bags closed with a paper clip and stored in a dry cabinet (Series FD Classic Line, Binder) at 70°C for 48 h. The dry weight and the water content (%) were measured as mentioned above.

### 2.5 Measurement of relative leaf chlorophyll content and stomatal conductance

The estimated chlorophyll content was measured regularly around noon starting on the day before the first stress period until the day before the last harvest. For relative chlorophyll content measurements, a Soil Plant Analysis Development (SPAD) chlorophyll meter (SPAD‐502 Plus, Konica Minolta, Germany) was used and the data of five similar spots of the oldest leaf above the cotyledons were collected and the average was used. Stomatal conductance of the oldest leaf above cotyledons was measured using a SC‐1 leaf porometer (Edaphic Scientific).

### 2.6 Measurement of leaf water potential using Scholander pressure bomb

The water potential of three plants per treatment was measured on the day after the second harvest. The water potential of the leaf was measured using a Scholander pressure bomb (Manometer, WIKA). As the plants were too big to fit in the pressure chamber, they were cut with scissors on top of the first leaf above the cotyledons. The upper plant part was hanging into the chamber and the plant was fixed and sealed with its stem sticking out. The cutting surface was freshly cut with a razor blade and then the pressure inside the chamber was slowly increased. As soon as water leakage was observed at the cutting surface, the current pressure in the chamber was recorded.

### 2.7 Determination of proline content in the leaf and the root

About 50 mg of fresh biomass of the leaf (the oldest leaf above the cotyledons) and roots were separately put in 2 ml Eppendorf tubes and immediately frozen in liquid N. After the harvest, they were stored in a freezer at −80°C. Examined were all samples of the second harvest. The determination of the proline content was performed according to a former study (Magne & Larher, 1992). For determination of proline content, 50 mg of dry leaf powder were extracted in 2 ml of 3% (w/v) sulfo‐salicylic acid (Magne & Larher, 1992). The analysis of the supernatant was done with two technical replicates for each sample. The dilutions for the calibration curve were measured in three technical replicates. All the solutions were prepared daily, and the calibration curve was repeated every day.

### 2.8 Determination of soluble sugars content in the leaf and the root

Soluble sugars were extracted using Anthrone Reagent (Galicia et al., 2009). Plant material analyzed in this test was the dried leaf and root samples. 20 mg dry mass was used except in some cases, where the root biomass was not enough and only 10 mg was used. This did not have any impact on the results, as the amount of water added was adapted. For the calibration curve, the sucrose dilutions were prepared in advance and stored frozen at −20°C. The calibration curve was repeated daily but without any technical replicates. Similar to the sample treatment, only 1 ml dilution was used for the colorimetric reaction. The rest of the procedure was carried out according to the mentioned protocol. The zero adjustment of the spectrophotometer was carried out with acrylic, disposable plastic cuvettes (Sarstedt) and Milli‐Q‐H2O (MILLI‐Q Synthesis System). As there were no replicates, samples with deviating results were either directly re‐measured (pseudo replicate) or completely re‐analyzed in the next run (repetition).

### 2.9 Statistical analysis

A completely randomized block design, with four replicates, was used to perform the experiment. By this design, we assured a random split of plants for drought treatment after the first harvest. Moreover, all the plants in the greenhouse had a random repositioning every day to minimize the position effects on the plant performance. Significant differences were calculated by Student's *t*‐test using excel 2016, in case of S1 and two‐way ANOVA using IBM SPSS 26, in the case of S2 and recovery. Statistical results (P‐values) obtained from Student's *t*‐test and two‐way ANOVA test are provided in Table S1.

### 2.10 RNA isolation and cDNA synthesis

For quantitative real‐time PCR (qRT‐PCR) analysis, total RNA was extracted from about 100 mg frozen leaf tissue (second leaf above cotyledon) using NucleoSpin RNA Plant and Fungi (Macherey‐Nagel), according to the manufacturer's instructions. RNA concentration was quantified photometrically using a NanoDrop (ND‐1000, Thermo Scientific). Complementary DNA (cDNA) synthesis was conducted on 2μg DNaseI‐digested total RNA using oligo‐(dT)18 and the RevertAid H Minus First Strand kit (Thermo Scientific).

### 2.11 qRT‐PCR primer design and assay

All the primers for qRT‐PCR have been previously reported (Najami et al., 2008). qRT‐PCR reactions were performed in 5 μl total reaction volumes including 2.5 μl Power SYBR Green Master Mix (ThermoFisher Scientific), 0.5 μM forward and reverse primers and 0.5 μl cDNA. *ACTIN* was used as reference gene. The thermal profile used for all qRT‐PCRs was: 10 min 95°C; (15 s 95°C; 1 min 60°C)40×. Data were analyzed by the 2^−ΔΔCt^ method (Schmittgen & Livak, 2008).

## 3 RESULTS

### 3.1 Optimization of drought stress duration

A trial series of different drought durations (2, 4, and 7 days) was conducted to study the effect of stress duration and choose the duration affecting sand and plant water content as well as plant dry biomass. It was observed that 4 and 7 days of drought decreased the above‐mentioned parameters, similarly, when compared to the control and 2 days drought (Figure S3a–c). Eventually, 4 days and not 7 days, of drought duration was chosen to have plants, which are not too dried out for further analysis.

To address the plants exposed to different treatments we have denoted abbreviations according to each stress and used those abbreviations to describe and discuss the results. The control plants receiving normal N and adequate water are denoted as C. Plants receiving normal N and drought are denoted as NND. Plants treated with low N but watered normally are denoted as LNW. Double stressed plants, receiving low N and drought are denoted as LND.3.2 A former Low N treatment did not affect the aboveground and root dry biomass in response to drought stress.

**FIGURE 1:**
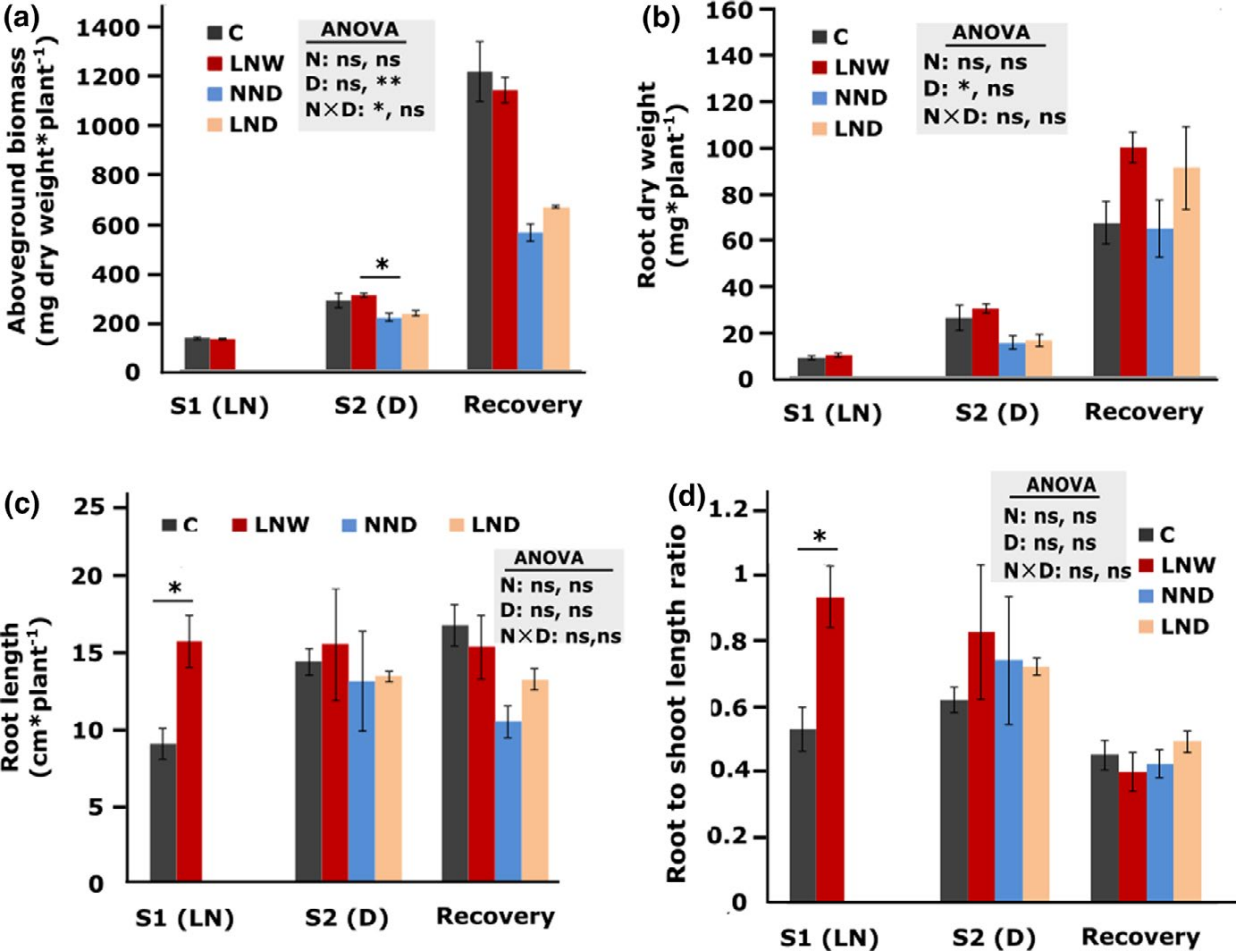
Effect of low N supply (LN) and drought (D) on the aboveground biomass (a), root biomass (b), root length (c), and root to shoot length ratio (d) of *Solanum lycopersicum*. The maximum root length was measured from the upper root branching to the tip of the longest root (maximum root depth). The shoot length was measured from the upper root to the branching of the youngest leaf. S1: first stress, S2, second stress. The different treatments are indicated as C: control, D: drought, W: watered normally, LN: low N supply (2 mM NO_3_^−^), NN: normal N supply (5 mM NO_3_^−^), combinations indicate the order of those treatments. Data represent means ± SD (n = 4). In case of S1 (LN) statistically significant difference between treatments was calculated using Student's *t*‐test (*p < 0.05; **p < 0.01; ***p < 0.001). In case of S2 (D) and recovery significant differences by N supply (N), drought (D) and interaction of the two parameters were calculated by two‐way ANOVA, represented in the gray box, for S2 (D) and recovery, respectively. Subsequently a Tukey's HSD post hoc test was done to identify significant differences between all different treatments, shown on the representative bars (*p < 0.05; **p < 0.01; ***p < 0.001)

The aboveground dry biomass (stem and leaves) of NND and LND plants, were lower in response to drought stress compared to the C plants (Figure 1a). The same was observed after the recovery period (Figure 1a).

It was observed that leaf number, and leaf dry weight decreased after drought stress, regardless of N treatment (Figure S4a,b). Moreover, NND and LND both increased specific leaf weight (Figure S4c). Moreover, stem dry weight was decreased after drought stress, regardless of N treatment (Figure S4d).

Root dry biomass did not show statistically significant differences between treatments. Only, the overall effect of drought was significant (*p* = 0.014) before the recovery phase (Figure 1b, Figure S4 (D) bars).

N‐deficiency in LNW plants resulted in significantly (*p* = 0.02) longer root compared to C plants before the first recovery phase. This effect was no longer observed when plants were exposed to drought stress and also one‐week recovery phase (Figure 3c).

LNW plants showed a significantly (*p* = 0.02) higher root to shoot ratio when compared to the control (Figure 1d). However, no significant differences were observed in root to shoot ratio between treatments after the first and the second recovery period (Figure 1d).

All in all, these data suggest that a former mild N‐deficiency does not necessarily have a negative effect on aboveground biomass, root dry weight and root length in response to subsequent drought stress.

### 3.2 Total water content and water potential were not affected by drought and N treatments

After drought treatment, plants showed no difference in total water content (Figure S5a) and water potential (Figure S5b), regardless of the former N treatment. In individual organs (leaf and stem), water content was decreased under drought regardless of N condition (NND and LND) (Figure S6a,b). This effect continued for the leaf even after the recovery period but not for the stem (Figure S6a,b). Root water content was not affected by drought or N treatment (Figure S6c).

### 3.3 Low N had a negligible effect on SPAD values while increased stomatal conductance under drought

**FIGURE 2:**
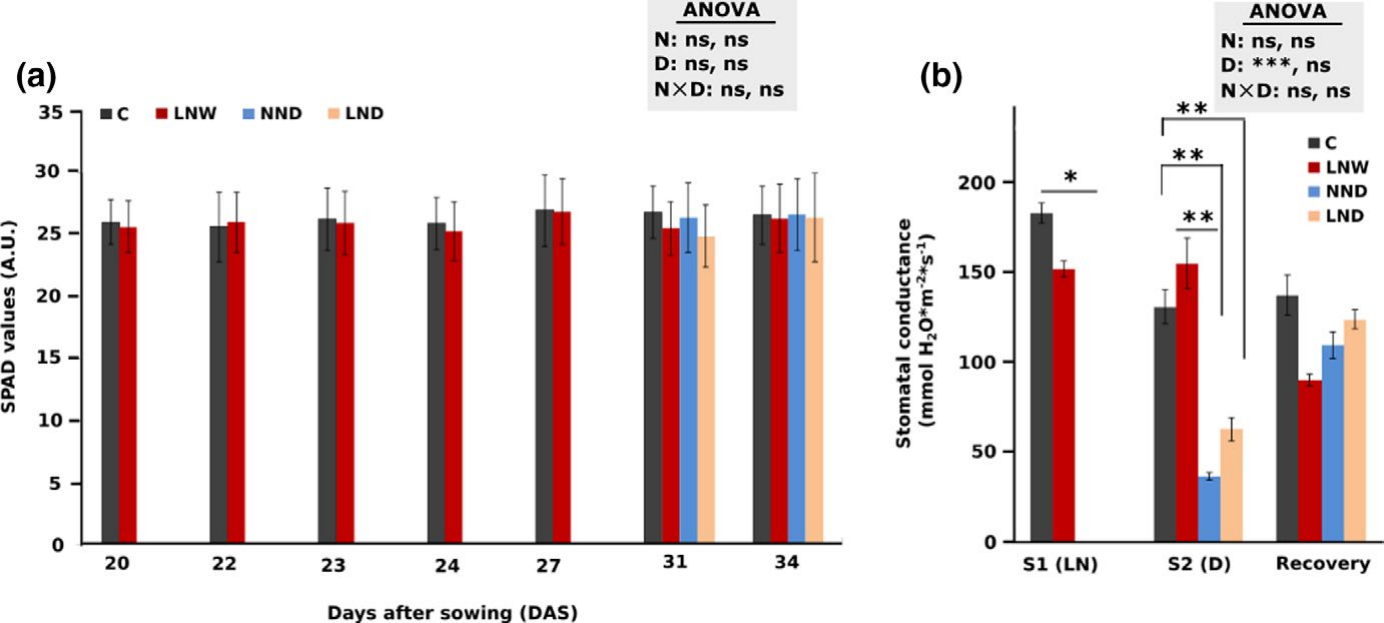
Chlorophyll content and stomatal conductance in the first leaf above the cotyledons of *Solanum lycopersicum*. Relative chlorophyll content (SPAD values) (a) and stomatal conductance (b) under each treatment were measured before noon. SPAD values were measured at three distinct points on the leaf (tip, left and right margin) and the average was taken as one biological replicate. S1: first stress, S2, second stress. The different treatments are indicated as C: control, D: drought, W: watered normally, LN: low N supply (2 mM NO_3_^−^), NN: normal N supply (5 mM NO_3_^−^), combinations indicate the order of those treatments. Data represent means ± SD (n = 4).). In case of S1 (LN) as well as SPAD values, at 20 to 27 DAS, statistically significant differences between treatments were calculated using Student's *t*‐test (*p < 0.05; **p < 0.01; ***p < 0.001). In case of S2 (D), recovery and SPAD values, at 31 and 34 DAS, significant differences by N supply (N), drought (D) and interaction of the two parameters were calculated by two‐way ANOVA, represented in the gray box, for S2 (D) and recovery, respectively. Subsequently a Tukey's HSD post hoc test was done to identify significant differences between all different treatments, shown on the representative bars (*p < 0.05; **p < 0.01; ***p < 0.001)

Although the mild N‐deficiency in this study was not severe enough to result in a major reduction in leaf N status and therefore chlorophyll loss, expressed in the SPAD values (Figure 2a), although it was effective on the other parameters examined such as increasing root length (Figure 1c), root to shoot ratio (Figure 1d) and leaf dry biomass (Figure S4a) when compared to the plants receiving adequate N.

The stomatal conductance was measured before each harvest. Four days after the drought period, the stomatal conductance was significantly reduced in drought‐treated plants under both N conditions (NND and LND) compared to C plants (*p* = 0.001 and *p* = 0.003, respectively) and N‐deficient LNW plants (*p* = 0.0013) (Figure 2b). After the one‐week recovery period, the values of formerly drought‐stressed plants (NND and LND) returned to approximately those of the C plants (Figure 2b). LNW plants showed lower stomatal conductance before the first recovery period and after the one‐week recovery period. (Figure 2b). It was noticeable that LND plants had a higher stomatal conductance (ca. 2‐fold) compared to NND plants, though not statistically significant after 4 days drought (Figure 2b), indicating the effect of a former N‐deficiency on stomatal conductance under drought stress.

### 3.4 Former N treatment affected osmolyte (soluble sugars and proline) content under drought stress

**FIGURE 3:**
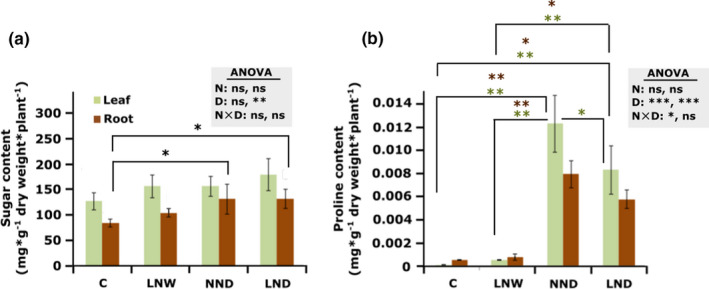
Effect of low N supply (LN) and drought (D) on the content of osmolytes. The amount of soluble sugars (a) and proline (b) was measured in the root and leaf of *Solanum lycopersicum* plants. The different treatments are indicated as C: control, D: drought, W: watered normally, LN: low N supply (2 mM NO_3_^−^), NN: normal N supply (5 mM NO_3_^−^), combinations indicate the order of those treatments. Data represent means ± SD (n = 4). Significant differences by N supply (N), drought (D) and interaction of the two parameters were calculated by two‐way ANOVA, represented in the gray box, for S2 (D) and recovery, respectively. Subsequently a Tukey's HSD post hoc test was done to identify significant differences between all different treatments, shown on the representative bars, green and brown stars represent significant differences in leaf and root, respectively (*p < 0.05; **p < 0.01; ***p < 0.001)

The content of soluble sugars and proline was detected in plant materials collected during the second harvest, after 4 days of drought stress. Leaf sugar content was not significantly affected by the different treatments (Figure 3a). Drought and double stress resulted in significantly higher soluble sugar content in the root of NND and LND plants compared to the C plants (*p* = 0.03 and *p* = 0.03, respectively) (Figure 3a). This indicates that a former mild low N treatment does not negatively affect the root osmolyte production, in the form of soluble sugars, in response to the subsequent drought stress.

Drought and double stress resulted in higher proline content in the root of NND and LND plants, compared to C plants (*p* = 0.005 and *p* = 0.003, respectively) and N‐deficient LNW plants (*p* = 0.03 and *p* = 0.03, respectively) (Figure 3b). In the leaf, drought only and double stress resulted in significantly higher proline level compared to control (*p* = 0.001 and *p* = 0.001, respectively) and N‐deficiency only (*p* = 0.001 and *p* = 0.001, respectively) (Figure 3b). This indicates that a former N deficiency might not have a negative impact on proline accumulation under drought stress.

### 3.5 Divergent expression response of *cAPX* genes under N‐deficiency and drought

Since the higher shoot biomass and root length of the LND plants were observed after one‐week recovery period (Figure 1a,c), the expression of cAPX genes (*cAPX1*, *cAPX2*, and *cAPX3*) was examined in the root and leaf after one‐week recovery.

**FIGURE 4:**
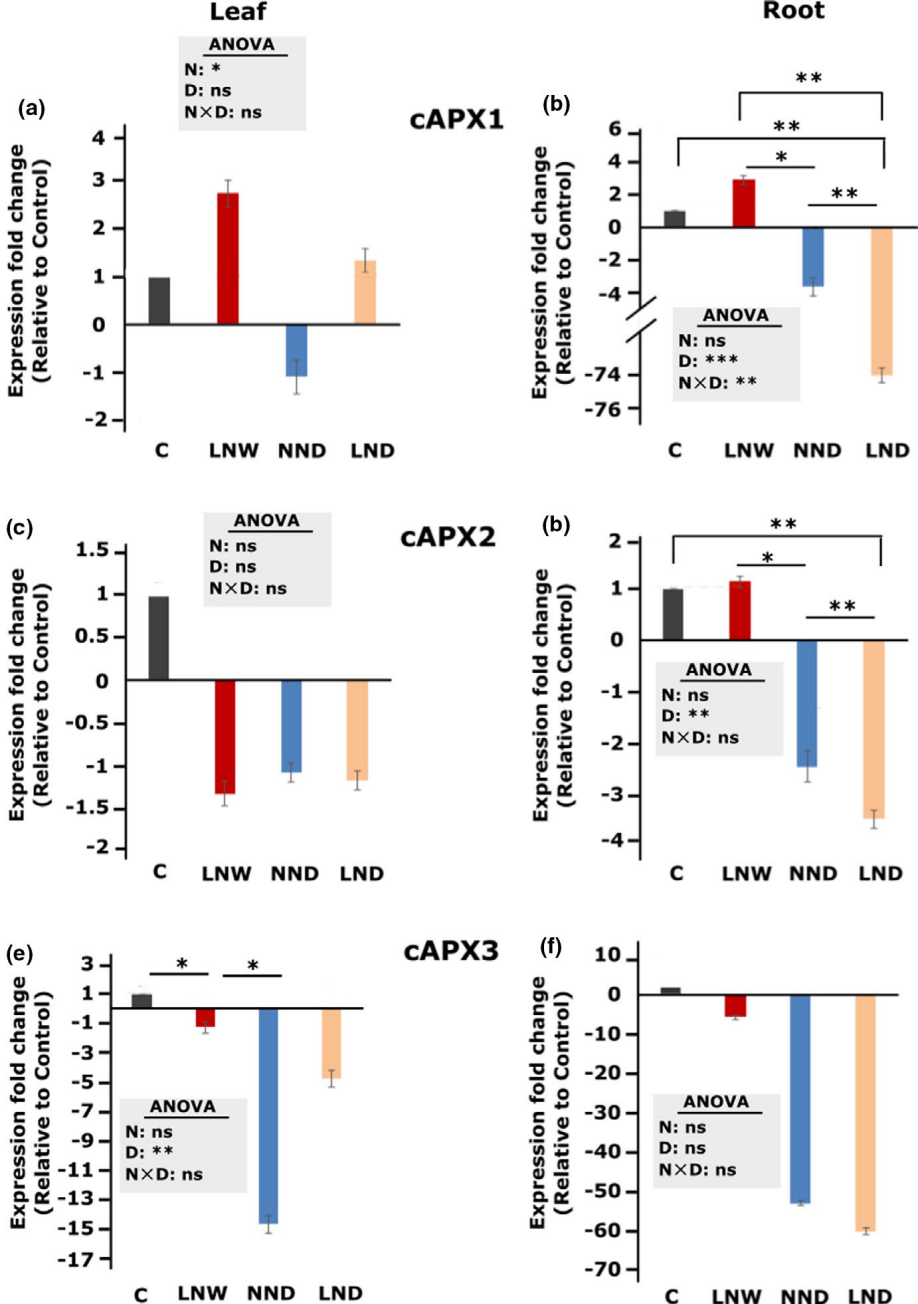
Effect of low N supply (LN) and drought (D) on the expression of *cAPX* genes in the root and leaf, after one‐week recovery period. *cAPX1* (a), *cAPX2* (b), and *cAPX3* (3) expression levels were detected in the root and leaf of *Solanum lycopersicum* plants using qRT PCR. The control expression level is set to 1 and the expression fold changes are shown relative to control. The different treatments are indicated as C: control, D: drought, W: watered normally, LN: low N supply (2 mM NO_3_^−^), NN: normal N supply (5 mM NO_3_^−^), combinations indicate the order of those treatments. Data represent means ± SD (n = 4). Significant differences by n: supply (LN), drought (D) and interaction of the two parameters were calculated by two‐way ANOVA, represented in the gray box, for S2 (D) and recovery, respectively. Subsequently a Tukey's HSD post hoc test was done to identify significant differences between all different treatments, shown on the representative bars (*p < 0.05; **p < 0.01; ***p < 0.001)

*cAPX1 and cAPX2* expression level in the leaf did not show any significant difference between different treatments (Figure 4a,c), although the overall effect of N was significant for *cAPX1* (*p* = 0.021). *cAPX3* showed downregulation in the leaf of LNW and NND plants compared to the control (Figure 4e). In the root, *cAPX1* and *cAPX2* showed upregulation in LNW compared to NND and LND (Figure 4b,d). It was noticeable that the lowest *cAPX1* and *cAPX2* expression was observed in the root of LND plants compared to all the other treatments (Figure 4b,d). *cAPX3* did not show any significant difference between different treatments in the root (Figure 4f).

## 4 DISCUSSION

The present study focuses on the effect of a former mild N‐deficiency on drought stress tolerance of tomato plants (cv. Moneymaker). To assess plant response to the stress, several morphological, physiological, and molecular analyses were assessed. Moreover, these analyses were conducted on root, leaf and, when possible, stem to dissect the organ‐specific stress response. To investigate the role of oxidative stress due to N‐deficiency, drought and their consecutive combination, the expression of the cAPXs genes which their encoded proteins function as cellular enzymatic antioxidants, and the amount of proline as a non‐enzymatic ROS scavenger were determined.

### 4.1 Mild low N did not have a negative impact on plant biomass under drought

Drought stress reduced aboveground (Figure 1a) and root dry biomass (Figure 1b) as well as root length in NND plants (Figure 1c). The reduction in growth has already been reported to be inevitable consequences of drought stress (Cruz de Carvalho, 2005). Our results showed that a former mild N‐deficiency did not enhance the negative effect of drought stress as no significant difference was observed between NND and LND plants in the measured morphological parameters (Figure 3a–d). Moreover, in the current study, the mild N‐deficiency increased root length significantly (Figure 1c) without inhibiting aboveground biomass (Figure 1a). This indicates that a higher root length under mild N‐deficiency (Figure 1c) might be beneficial for aboveground growth in response to the subsequent drought stress.

It has been reported that low N treatment has negative effects on plant growth (Toth et al., 2002) and yield (Warner et al., 2004). With N being an essential constituent of many macromolecules, the plant is inhibited in biomass production when there is not enough N available (Lupini et al., 2017). Despite the negative impact on shoot growth, N deficiency has been reported to induce primary root growth which facilitates N uptake and growth (Xu et al., 2019). The main N form in the soil is nitrate and ammonia which are both highly mobile and can easily be leached to the lower soil layer and out of the reach for plants (Xu et al., 2019). Root promotion under N deficiency is therefore a crucial response for better N uptake (Linkohr et al., 2002; Lynch, 2013). High N decreases root growth which minimizes root foraging for water and therefore has a negative effect on long‐term drought stress tolerance (Mi et al., 2008).

High N prevents plants from developing stress avoidance (Claeys & Inze, 2013). This is in accordance with reports showing that N fertilization minimizes plant performance under drought stress (Patterson et al., 1997).

An adverse interaction between drought and nutrient availability on the above‐ and belowground growth, photosynthesis, and transpiration has been reported (Kono et al., 1987). Indeed, N application can lead to a rapid development of drought stress in rice so that plants receiving high N were more sensitive to drought (Araus et al., 2020; Prasertsak & Fukai, 1997). It has been shown that N application has a negative effect on drought tolerance of lowland rice so that the maximum reduction of dry biomass could be observed under moderate drought and high N fertilization (Araus et al., 2020; Gong et al., 2010; Menge et al., 2019).

A recent study, investigated the performance of two contrasting wheat cultivars, Slomer and Enola, under N‐limitation. Slomer is an old tall cultivar while Enola is a modern semi‐dwarf cultivar (Kocheva et al., 2020). It was observed that Slomer performed better under N‐limitation in case of photosynthetic capacity, N‐assimilation (higher amino acid synthesis) and overall performance (Kocheva et al., 2020). Authors suggested that since selection of old verities has been conducted under low N condition, those varieties are more robust in response to limited N condition as they have a better ability to retained higher amino acids levels while modern varieties accumulate sugars, as it was observed in their study (Kocheva et al., 2020).

The effect of proper N management on plant drought tolerance has already been studied in Kentucky bluegrass during field trials (Saud et al., 2017). It was shown that drought stress results in a reduction of several physiological and morphological parameters such as N content, maximum radius, above and below‐ground biomass, number of ramets per plot, leaf water contents, and water potential. Moreover, drought increased the C/N ratio due to higher C content (Saud et al., 2017). The authors observed that proper nutrient management had a positive effect on ameliorating the negative consequences of drought stress on plant performance (Saud et al., 2017).

These data emphasize on the importance of better understanding of the molecular mechanisms involved in root growth under low N, particularly in important crops. Developing cultivars with better ability for nitrate and water uptake, through root induction, minimizes the N fertilizer application and might be beneficial for the tolerance against upcoming stresses such as drought.

### 4.2 Mild N‐deficiency enhanced stomatal conductance and osmolyte content under drought stress

Drought stress decreased stomatal conductance regardless of N treatment (Figure 2b). Lower stomatal conductance during the stress period and lower carbon (C) fixation, decreases the nicotinamide adenine dinucleotide phosphate (NADPH) consumption by the Calvin cycle and therefore less NADPH molecules and more atmospheric oxygen are available as electron acceptors (Ben Rejeb et al., 2014). This leads to the formation of cell‐damaging ROS molecules (Ben Rejeb et al., 2014).

Proline accumulation in this study was negatively correlated with water content (%). So that, after the second harvest, the NND and LND plants showed the highest proline content in both leaf and root (Figure 3b) while the water content (%) in those samples was lower compared to the control and N‐deficient plants (Figure S5a). A similar negative correlation between proline level and relative water content has been observed in several lotus species (Diaz et al., 2005).

Although the osmo‐protective role (lowering the cellular water potential and thus avoid water withdrawal to the apoplast) is widely reported, it has been recently questioned how substantially this function can be applied to water stress (Forlani et al., 2019; Sarker et al., 2005). A relatively low absolute concentration of accumulated proline has been observed in several cases, which was considered too low to cause enough reduction of the water potential (Forlani et al., 2019). Therefore, the authors suggested a more significant role for proline as a stabilizer for enzymes and membranes by maintaining the macromolecules’ hydration shell rather than a role as an osmo‐protectant (Forlani et al., 2019).

Proline is also considered to be involved in ROS scavenging (Forlani et al., 2019; Liang et al., 2013). Proline is therefore important for the oxidative stress resulting from drought. Thus, proline has several functions in the cell such as osmoregulation, stabilization of macromolecules, buffering of the cellular redox potential and scavenging of ROS. It has also been proposed that the biosynthesis of proline from glutamate plays a role in the limitation of ROS generation (Ben Rejeb et al., 2014; Hayat et al., 2012; Tripathi & Gaur, 2004).

Proline synthesis requires two NADPH molecules which leads to the production of oxidized NADP^+^, which in turn can be used as an electron acceptor in the photosynthesis (Ben Rejeb et al., 2014). This is in line with a higher level of proline in our study in response to drought and double stress, indicating its potential in osmo‐regulation and possibly protection from oxidative stress.

As expected, while proline content of the LND plants was slightly lower compared to NND (Figure 3a), the root sugar content was significantly higher in LND plants compared to the control, possibly due to the higher C/N level (Figure 3b). Higher amounts of soluble sugars might have a compensation effect for the slightly reduced amount of proline in LND plants under drought. However, it remains to be determined whether proline accumulation in LND plants is more likely the result of increased *de novo* biosynthesis or greater protein degradation.

### 4.3 Higher *cAPX1* expression in N‐deficient and double‐stressed plants after recovery period indicates its involvement in potential redox protection

ROS generation in response to N‐deprivation has already been reported (Shin et al., 2005). In Arabidopsis roots, NADPH oxidases might be involved in H_2_O_2_ production in response to N‐deficiency (Kwak et al., 2003; Shin et al., 2005). Removal of root hairs led to ROS reduction in Arabidopsis root under N‐deficiency. Therefore, it has been suggested that root hairs might play a role in N‐deficiency sensing and response (Shin et al., 2005).

Drought stress results in the accumulation of ROS affecting plant growth and development (Lee & Park, 2012; Qi et al., 2018; Thirumalaikumar et al., 2018; You & Chan, 2015). High ROS level, caused by a disturbance of the balance between ROS production and scavenging, results in oxidative stress which in turn leads to various negative effects on plant growth and development (Lee & Park, 2012).

The expression of the genes encoding the enzymes involved in the ascorbate‐glutathione cycle is shown to be responsive to drought duration and intensity. While c*APX1* expression is constitutive, *cAPX2* expression is induced in response to high light, heat stress, or wounding (Koussevitzky et al., 2008). Moreover, the redox state of the photosynthetic electron transfer chain influences the expression of both *cAPX*1 and *cAPX2* genes (Karpinski et al., 1997). cAPX1 has been shown to protect chloroplasts against ROS so that the absence of cAPX1 leads to a drastic elimination of chloroplast ROS scavenging machinery leading to higher H_2_O_2_ level and protein oxidation (Davletova et al., 2005). It has been shown that ascorbate level increased after short‐term low N treatment in cucumber (Zhao et al., 2015). In tea plant, AsA metabolism under N‐deficiency is shown to be regulated through the interaction of cAPX1 with N regulatory protein P‐II (GLB1) (Li et al., 2020). Overexpression of celery *cAPX1* gene in Arabidopsis, improved drought resistance and AsA accumulation in transgenic plants (Liu et al., 2019). Moreover, drought stress is shown to decrease AsA level in soybean plants (Seminario et al., 2017).

In the current study, strong higher expression of *cAPX1* under N‐deficiency (LNWand LND) (Figure 4) suggests that *cAPX1* plays a role in the acclimation of plants to consecutive drought and N‐deficiency, after a one‐week recovery period. Down‐regulation of *cAPX* genes which was observed in all treatments (except for cAPX1 in LNW and LND) might be associated with a lower level of AsA under drought stress. However, a higher expression in the time before recovery cannot be excluded, as it was not investigated here. More investigations are required to study the role of the antioxidative system under consecutive N‐deficiency and drought stress.

One of the limitations of this study is that, before the second harvest, drought treated plants (NND and LND) did not receive the nutrient solution, but this was not the case for other treatments (C and LNW). Therefore, it is difficult to distinguish between the effect of drought and N‐deficiency in our study during the second harvest. Moreover, investigating the effect of a reverse order in the stresses, by applying drought as the first stress and N‐deficiency as the second stress, is required to give a more complete view of the effect of these two stressors and their orders on the plant performance.

The effect of N metabolism efficiency on combined stress tolerance has already been investigated. For instance, two recombinant inbred lines (RIL‐66 and RIL‐76) from a cross between *Solanum lycopersicum* and *Solanum pimpinellifoilum* with contrasting tolerance to the combination of salinity and heat were investigated (Lopez‐Delacalle et al., 2020). When subjected to the combination of salinity and heat stress, the tolerant line (RIL‐76) showed a higher level of total protein and amino acid content compared to the sensitive line (RIL‐76) (Lopez‐Delacalle et al., 2020). Moreover, glutamate and glutamine level as well as expression of nitrogen metabolism‐related genes such as NITRATE REDUCTASE (*NR*), *NITRITE REDUCTASE* (*NiR*), *GLUTAMATE DEHYDROGENASE* (*GDH*), *GLUTAMINE TRANSPORTER 1* (*GLT1*), *NITRATE TRANSPORTER 1*.*2* (*NRT1*.*2*), *AMMONIUM TRANSPORTER 1* (*AMT1*), and *AMMONIUM TRANSPORTER 2* (*AMT2*) were also higher in RIL‐76 (Lopez‐Delacalle et al., 2020). This indicates the importance of an efficient nitrogen metabolism for combined stress tolerance. Due to the climate change and frequent encounters of the plants to various stress scenarios, more investigations are required to understand the effect of nitrogen‐related metabolism on the response of plants to cross‐stress conditions.

## 5 CONCLUSION

**FIGURE 5:**
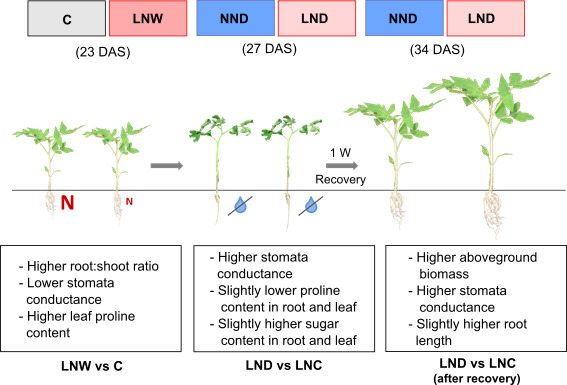
Schematic model illustrating the main changes observed in measured parameters in response to low N supply and adequate water or low N supply and drought in tomato plants. Plants were treated with low N or normal N supply before drought stress. Different treatments are indicated as C: control, D: drought, W: watered normally, LN: low N supply (2 mM NO_3_^−^), NN: normal N supply (5 mM NO_3_^−^), combinations indicate the order of those treatments. DAS: days after sowing

In summary, our results indicate that a short mild N‐deficiency by reducing N fertilizer might be beneficial for minimizing the negative effects of drought stress. However, the intensity of the N‐deficiency needs to be carefully examined to prevent yield loss due to low N. Cross‐stress tolerance is a promising approach to reduce both water and N inputs with high relevance for supplying the growing world population with food in times of climate change. Thus, further research, for instance, analyzing different stress durations, crops, and developmental stages is necessary for agriculture application. Moreover, studying the response of N‐efficient and N‐inefficient cultivars as well as drought tolerant and sensitive cultivars can shed more light to the cross‐stress tolerance of N‐deficiency and drought in the field. Another exciting aspect would be investigating the impact of N‐deficiency on the soil‐ and plant‐microbiome and the effects of the modified plant–microbe interactions in response to drought. It is noteworthy to mention the current study is conducted in the greenhouse under a controlled condition and therefore, it might not represent the most simulated environment in the real world. Therefore, further investigations using crops in the field is required to confirm the results of this study in the context of climate change. A schematic model (Figure 5) is illustrated which summarizes the differences detected between single and double‐stressed plants in this study. Further investigations are required to unravel the underlying molecular mechanisms involved in plant adaptation response to drought after being exposed to contrasting N supplies.

## CONFLICT OF INTEREST

The authors declare no competing interests.

## AUTHORS’ CONTRIBUTION

V.S.R. Conceived, designed, co‐performed in physiological experiments and as well as gene expression assays and wrote the paper. K.U. Performed the physiological measurements and analyzed the data and created the diagrams. B.Ö. Performed the gene expression assays, analyzed the data and created the diagrams and reference management. H.S.R Performed the statistical analysis and edited the diagrams according to the statistical data. C.S. Conceived the experiment and edited the paper.

## Supporting information

Fig S1

Fig S2

Fig S3

Fig S4

Fig S5

Fig S6

Table S1

## Data Availability

The data that support the findings of this study are available in the supplementary material of this article.
