# Supplementary figures and images for: Cross‐stress tolerance: Mild nitrogen (N) deficiency effects on drought stress response of tomato (Solanum lycopersicum L.)
Source: Plant Environ Interact. 2021 Oct 7;2(5):217–28. doi: 10.1002/pei3.10060 (PMC10168089; doi:10.1002/pei3.10060)

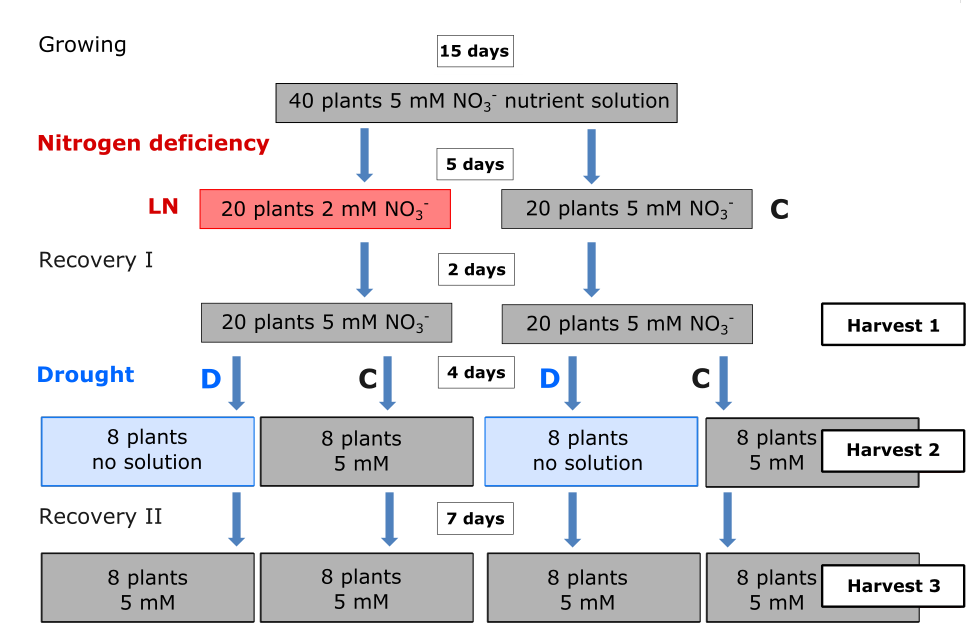

Supplement: Supplementary file 1 — Fig S1 [file PEI3-2-217-s001.png]

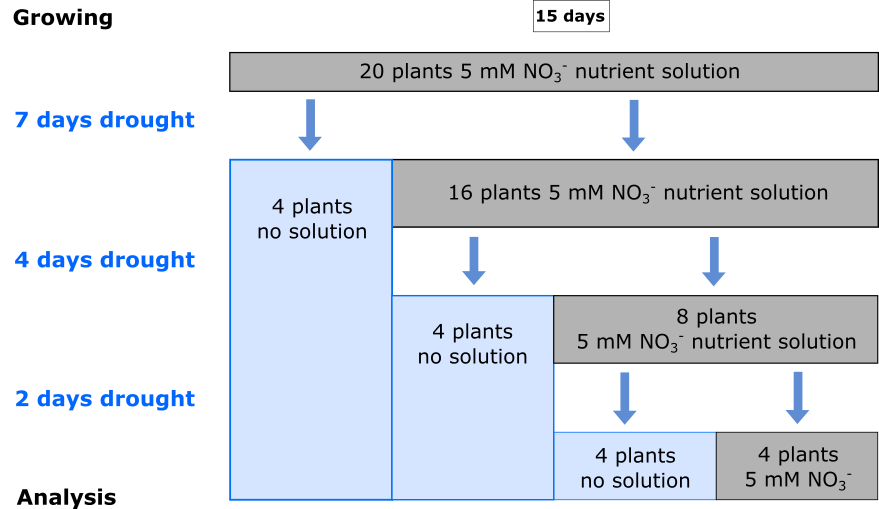

Supplement: Supplementary file 2 — Fig S2 [file PEI3-2-217-s002.png]

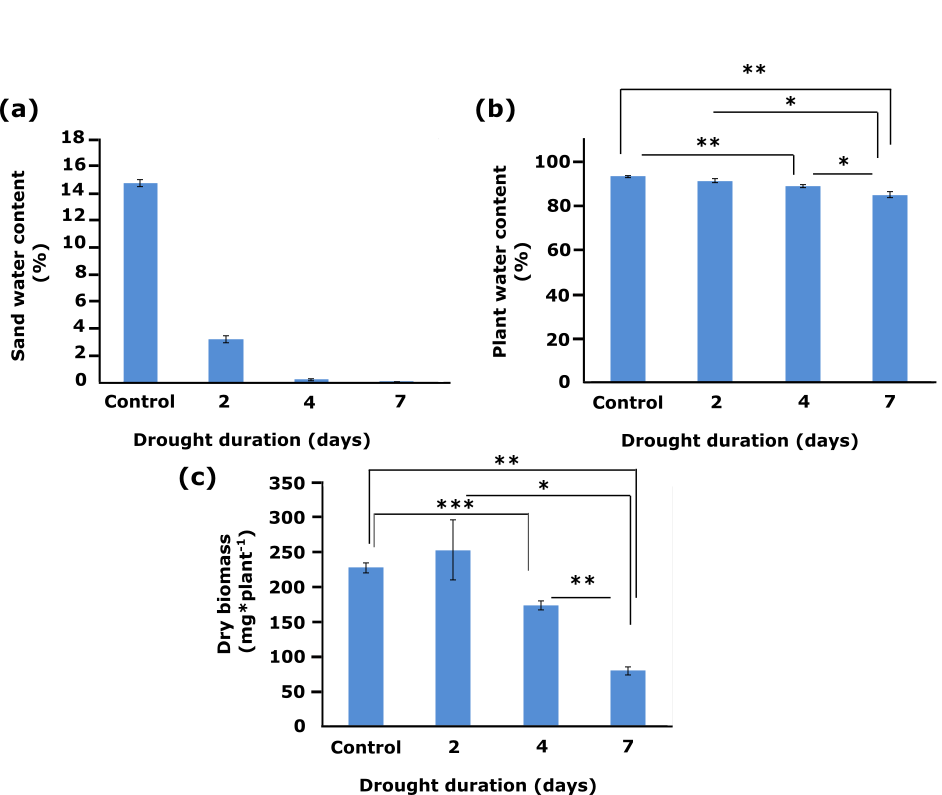

Supplement: Supplementary file 3 — Fig S3 [file PEI3-2-217-s006.png]

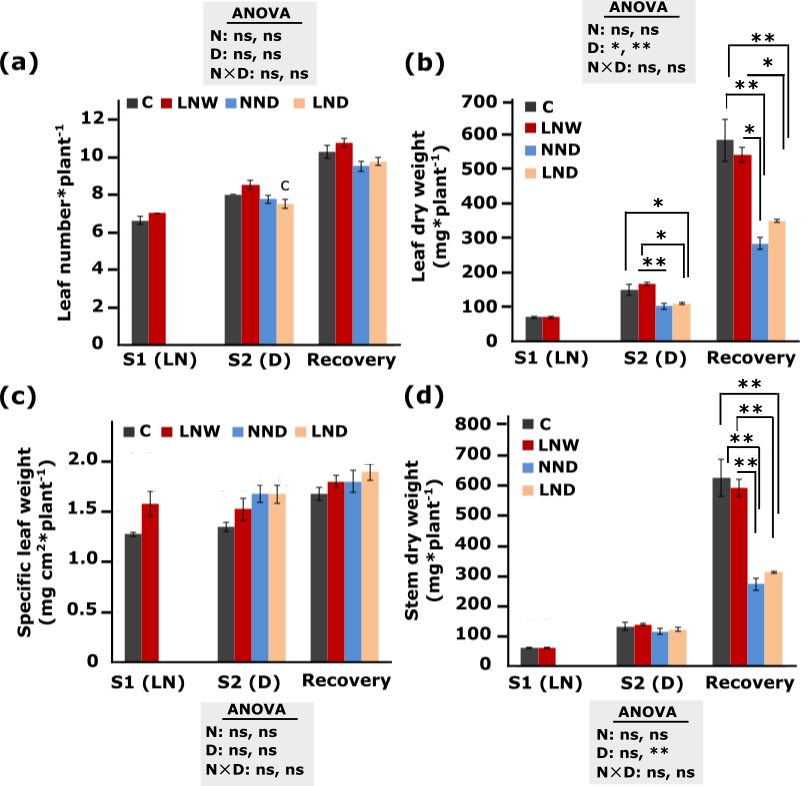

Supplement: Supplementary file 4 — Fig S4 [file PEI3-2-217-s003.png]

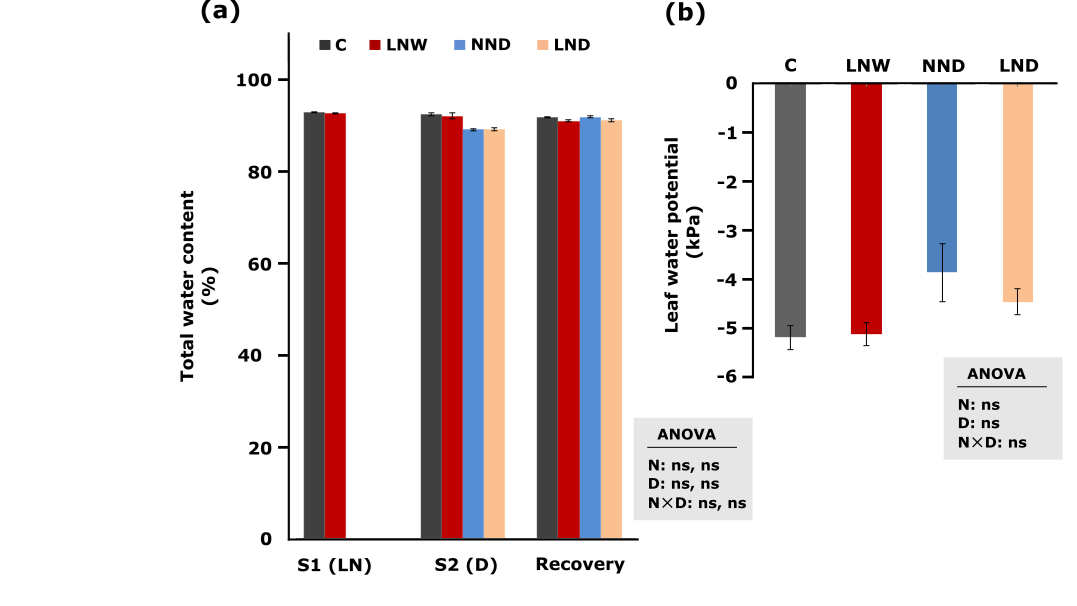

Supplement: Supplementary file 5 — Fig S5 [file PEI3-2-217-s005.png]

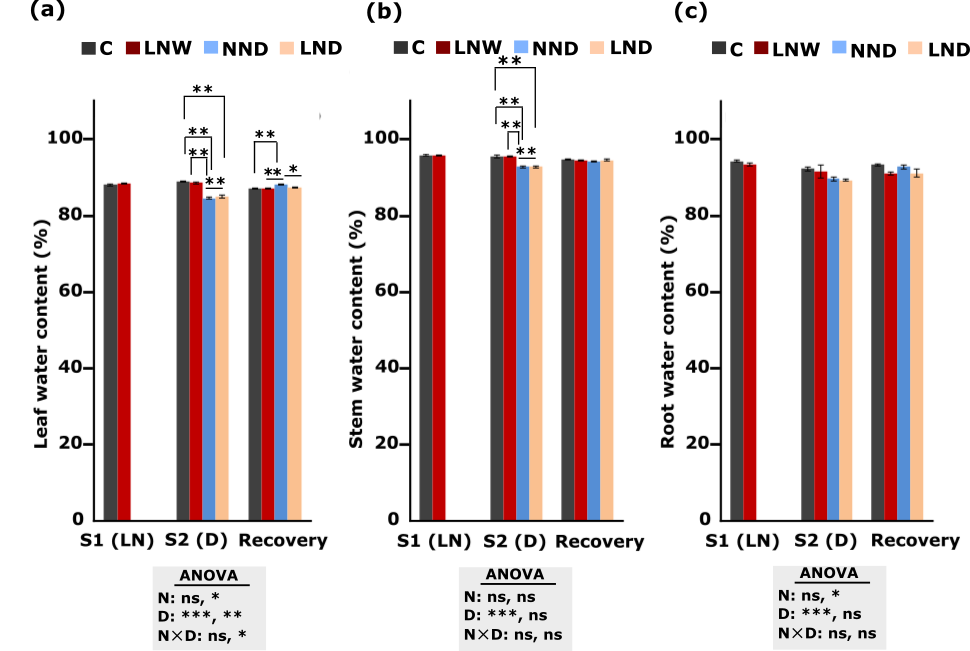

Supplement: Supplementary file 6 — Fig S6 [file PEI3-2-217-s004.png]
